# The Protective Effects and Mechanisms of Apelin/APJ System on Ischemic Stroke: A Promising Therapeutic Target

**DOI:** 10.3389/fneur.2020.00075

**Published:** 2020-03-03

**Authors:** Yanjun Tian, Ruijiao Chen, Yunlu Jiang, Bo Bai, Tongju Yang, Haiqing Liu

**Affiliations:** ^1^Collaborative Innovation Center for Birth Defect Research and Transformation of Shandong Province, Jining Medical University, Jining, China; ^2^School of Mental Health, Jining Medical University, Jining, China; ^3^Institute of Neurobiology, Jining Medical University, Jining, China; ^4^Department of Pharmacy, People's Hospital of Zoucheng City, Jining, China; ^5^Department of Physiology, Shandong First Medical University (Shandong Academy of Medical Sciences), Taian, China

**Keywords:** apelin/APJ system, ischemic stroke, signaling pathways, neuroprotection, molecular mechanisms

## Abstract

The orphan receptor APJ and its endogenous ligand apelin, which are expressed in the brain, are the major components of the apelin/APJ system. Growing evidence shows that the apelin/APJ system plays a vital role in the pathophysiology of cerebral ischemic injury. Targeting the apelin/APJ system may have protective effects on cerebral ischemic injury. In this review, we sum up the latest research progress relating to the actions and therapeutic potential of the apelin/APJ system in ischemic stroke. An in-depth knowledge of the pathophysiological effects of the apelin/APJ system and the underlying mechanisms will help to develop novel therapeutic interventions for ischemic stroke.

## Introduction

In 1993, O'Dowd discovered the orphan G protein–coupled receptor (GPCR) APJ while searching for vasopressin receptor subtypes (1). APJ is encoded by a gene located on chromosome 11q12. Although APJ shares 54% homology with angiotensin II receptor type-1 (AT1R) in the hydrophobic transmembrane region, there's no binding site for angiotensin II (1). Tatemoto et al. isolated apelin, the cognate ligand for APJ receptor, from bovine stomach tissue extracts in 1998 (2). The apelin gene, which is located on chromosome Xq25-26.1, encodes the preproapelin of 77 amino acids (apelin-77). Various bioactive isoforms of apelin are derived from apelin-77, including apelin-55, apelin-36, apelin-17, apelin-13, and apelin-12 (3, 4). Recently Chng et al. discovered apela, another endogenous ligand for APJ, which is encoded by a gene located on chromosome 11 and is critical in embryonic development (5, 6). However, in humans, the apela is only expressed in pluripotent cells and kidney (7). The apelin/APJ system mainly refers to APJ and its endogenous ligand, apelin.

Stroke, which is mainly caused by cerebral vascular occlusion and cerebral blood supply disorder, is one of the leading causes of death and disability worldwide, and 87% of cases are ischemic stroke (8). The cerebral infarction area is composed of the ischemic core and penumbra; apoptosis is the main cause of neuronal damage in the penumbra region, which also provides an opportunity for the treatment of ischemic stroke and makes it possible to use drugs to alleviate neuronal injury since the apoptosis is delayed and reversible (9, 10). Neuronal apoptosis in ischemic penumbra is triggered by diffusion of toxic substances released by the dead neurons of the ischemic core in the acute stage of ischemia, while ischemia injury is aggravated after reperfusion, namely ischemia/reperfusion (I/R) injury, which contributes to the neuron apoptosis in the penumbra via numerous biological mechanisms, including excitotoxicity, oxidative and nitrative stress, inflammatory responses, endoplasmic reticulum stress (ERS), and so on (11–14).

The apelin/APJ system is widely expressed in the central nervous system, especially in neurons and oligodendrocytes (15, 16). Growing evidence indicates that the apelin/APJ system is involved in the pathophysiology of ischemic stroke (17, 18). Targeting the apelin/APJ system may have protective effects on cerebral ischemic injury. In this review, we mainly focus on the latest research progress related to the biological functions and therapeutic potential of the apelin/APJ system in ischemic stroke.

## Cellular Signaling Pathways of APELIN/APJ System

**Figure 1:**
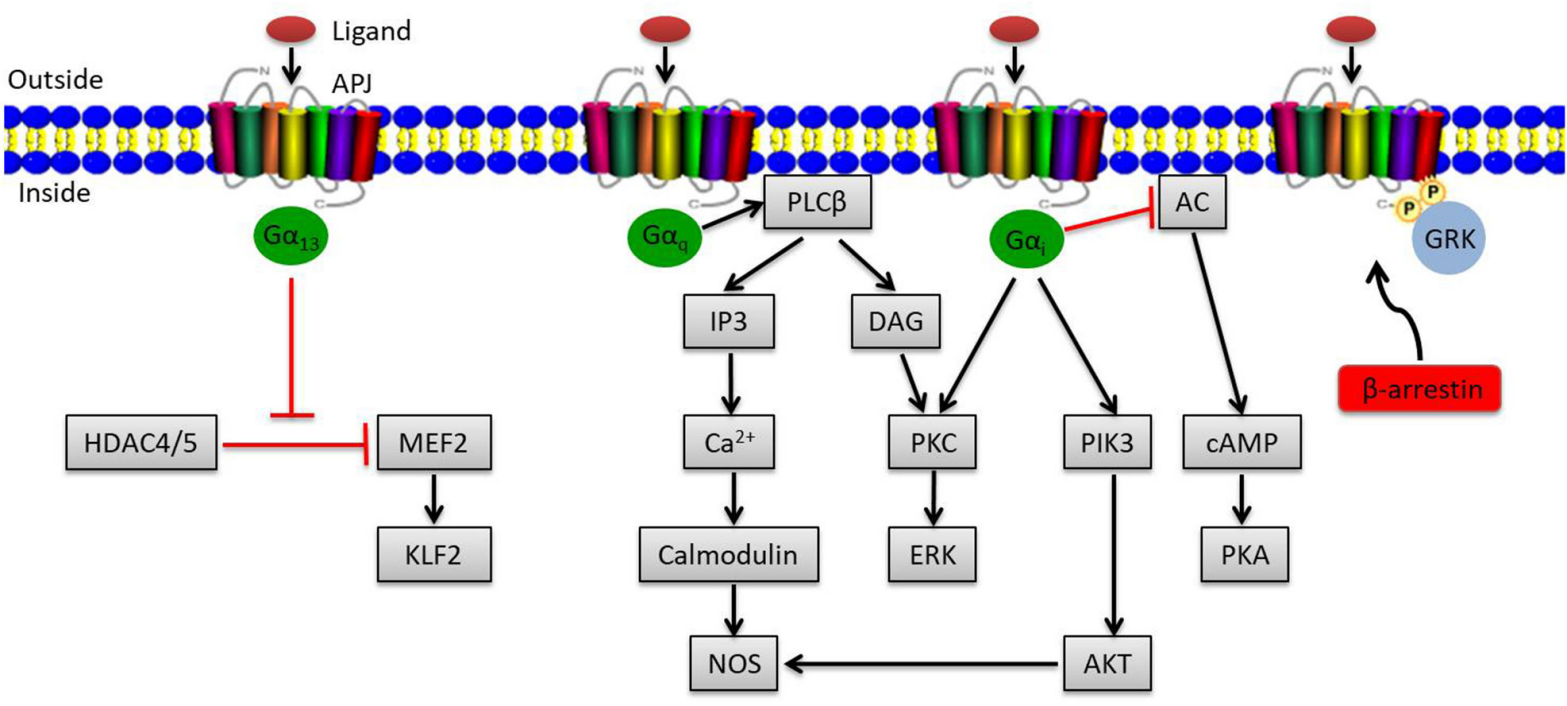
Overview of apelin/APJ system–mediated signaling pathways. Canonical ligand-dependent APJ signaling via Gα_i_ and Gα_q_ leads to the activation of protein kinase C (PKC), phosphatidylinositide 3-kinase (PI3K), and nitrous oxide synthase (NOS) pathways and the inhibition of adenylyl cyclase (AC) (19). In endothelial cells, ligand activates a Gα_13_-dependent pathway that allows the transcription of myocyte enhancer factor-2 (MEF2). Moreover, the G protein–independent pathway of the apelin/APJ system is mediated by G protein–coupled receptor kinase (GRK) and β-arrestin. Arrowheads indicate activation, and blunted arrows indicate inhibition.

The apelin/APJ system mediates signal transduction mainly by coupling to G protein (Figure 1). Gα_i_ participates in the activation of phosphatidylinositide 3-kinase (PI3K)/protein kinase B (PKB, also known as AKT) or contributes to protein kinase C (PKC) activation, initiating the mitogenic extracellular signal-regulated kinase (ERK) signaling pathway; it can also inhibit the activation of protein kinase A (PKA) by inhibiting adenylyl cyclase (AC) to produce cyclic adenosine monophosphate (cAMP) (19, 20). Meanwhile, Gα_q_ activates phospholipase C beta (PLCβ), inducing the production of diacylglycerol (DAG) and inositol 1,4,5-triphosphate (IP3), facilitating the activation of PKC cascade and the release of intracellular Ca^2+^ respectively (19). Ca^2+^ release in turn activates calmodulin, which subsequently exerts vasodilatory effects via activating nitrous oxide synthase (NOS). Besides that, endothelial NOS (eNOS) can be induced by AKT activation via Gα_i_, also presenting a vasodilation effect of the apelin/APJ system (21). In addition to the canonical pathways, Kang et al. found a novel APJ signaling route in endothelial cells through Gα_13_, leading to phosphorylation and cytoplasmic translocation of class II histone deacetylases (HDACs) 4 and 5, thereby activating myocyte enhancer factor-2 (MEF2), which induces the expression of MEF2 target gene Kruppel-like factor 2 (KLF2) (Figure 1) (22).

Apart from the canonical G protein–dependent pathways, stimulation of APJ by apelin leads to phosphorylation of APJ via G protein–coupled receptor kinase (GRK) and the subsequent recruitment of β-arrestin, causing desensitization and internalization of APJ, which can activate G protein–independent signaling pathways (Figure 1) (23).

All apelin isoforms share the same 12 C-terminal residues (RPRLSHKGPMPF), which are required for bioactivity (24–28). The smaller apelin isoforms play a unique role in activating certain downstream signaling of APJ, which often induces APJ to be quickly recycled back to the cell surface, while the larger ones with higher affinity for APJ usually account for increased intracellular retention or even degradation (20, 29).

## Expression Changes of APELIN/APJ System in Ischemic Stroke

The expression of the apelin/APJ system components in different stages of ischemic stroke is temporally altered (18). A number of transcription factors, including Sp1 transcription factor (SP1), hypoxia inducible factor 1 alpha (HIF-1α), activating transcription factor 4 (ATF4), signal transducer and activator of transcription 3 (STAT3), and so on, are involved in regulating the expression of the apelin/APJ system (30–34).

Oxygen and glucose deprivation are the main consequences of ischemia, which are related to the abnormal expression of the apelin/APJ system (32, 35). During cerebral ischemia phase, the expression of the apelin/APJ system is upregulated, which is induced by HIF-1α and SP1 (30–32). Growing evidence shows that HIF-1α can bind to hypoxia response elements located in the promoter region of apelin and APJ genes under hypoxic conditions, which then significantly enhances the expression of the apelin/APJ system (32, 36). Moreover, the expressions of apelin and APJ in neurons in the early stage of ischemia are induced by increased SP1, which is possibly mediated by HIF-1α (30, 31, 37, 38).

However, the apelin/APJ system expression is downregulated during the reperfusion phase. Compared with the normoxia control group, the expression of APJ in the mouse hippocampus is significantly reduced after 4 weeks of chronic normobaric hypoxia treatment, which can be reversed by apelin-13 (39). Oxidative stress, ERS, autophagy, and inflammatory responses and the interaction between them may be related to the downregulated expression of the apelin/APJ system (18, 40, 41). For example, ERS, which is activated only during the reperfusion phase rather than the ischemic phase, plays critical roles in cerebral I/R injury (42–44). As an important regulator of ERS, ATF4 negatively regulates the expression of the apelin gene via the pro-apoptotic p38 mitogen-activated protein kinase (MAPK) pathway, which indicates that ERS may promote the repression of apelin at the reperfusion stage (34). Consistently, one recent study showed that apelin-12 could reduce neuron apoptosis of the ischemic penumbra in middle cerebral artery occlusion (MCAO)–induced ischemic mice by inhibiting the C-Jun N-terminal kinase (JNK) and P38MAPK signaling pathways (45).

To sum up, the expression of the apelin/APJ system in ischemic stroke is altered in different phases with complex mechanisms, indicating that targeting the apelin/APJ system may provide novel therapeutic interventions for ischemic stroke.

## Neuroprotection of APELIN/APJ Signaling in Ischemic Stroke and the Underlying Mechanisms

Recently, numerous studies show that the apelin/APJ axis possesses neuroprotective effects by inhibiting neuronal apoptosis and improving functional recovery through diverse pathways in ischemic stroke (Table 1).

**Table 1:** Effects of apelin/APJ signaling on ischemic stroke.

**Experimental model**	**Pathway**	**Effect**	**References**
Mice *in vivo* (MCAO)	↑PI3K/AKT, ↑ERK	Protection	(46, 47)
Rats *in vivo* (MCAO)	↑ERK	Protection	(48)
Mice *in vivo* (MCAO)	↑AMPK	Protection	(49)
Rats *in vivo* (MCAO)/OGD/R cell model neurons	↑Gα_i_/Gα_q_-CK2, ↓eIF2-ATF4-CHOP	Protection	(14)
Rats *in vivo* (MCAO)	↓ERS/UPR	Protection	(13)
Mice *in vivo* (MCAO)/neonatal H/I injury rat model	↑PI3K/AKT	Protection	(50)
Mice *in vivo* (MCAO)	↓JNK, ↓P38MAPK	Protection	(45)
Rats *in vivo* (MCAO)/OGD/R cell model neurons	↑VEGF–VEGFR2, ↑ERK, ↑PI3K/AKT	Protection	(51)
Rats *in vivo* (MCAO)/PC12 cells	↑AMPK/GSK-3β/Nrf2	Protection	(52)

*MCAO, middle cerebral artery occlusion; P13K, phosphatidylinositide 3-kinase; ERK, extracellular signal-regulated kinase; AMPK, AMP-activated protein kinase; CK2, casein kinase 2; OGD/R, oxygen-glucose deprivation/reperfusion; ATF4, activating transcription factor 4; CHOP, CCAAT/enhancer binding protein homologous protein; ERS, endoplasmic reticulum stress; UPR, unfolded protein response; H/I, hypoxia/ischemia; JNK, C-Jun N-terminal kinase; P38MAPK, p38 mitogen-activated protein kinase; VEGF, vascular endothelial growth factor; VEGFR, VEGF receptor; GSK-3β, glycogen synthase kinase 3 β; Nrf2, nuclear factor erythroid 2–related factor 2*.

### Blocking Excitotoxicity

Excitotoxicity occurs immediately after the onset of ischemia. During the excitotoxic phase with energy being depleted, membrane potential is lost, and neurons and glia depolarize, which is followed by the activation of somatodendritic and presynaptic voltage-dependent Ca^2+^ channels and the diffusion of excitatory amino acids in the extracellular space (53, 54). Meanwhile, the extracellular accumulation of excitatory amino acids (especially glutamate) was further increased, since the presynaptic reuptake of excitatory amino acids is obstructed, leading to over-activation of two distinct ionotropic receptors, namely the N-methyl-D-aspartate (NMDA) receptor and the α-amino-3-hydroxy-5-methyl-4-isoxazole (AMPA) receptor, which facilitates an excessive Ca^2+^ influx into neurons and initiates neuronal damage or death (55).

Substantial research showed that apelin could protect neurons against excitotoxicity induced by quinolinic acid (QUIN) and HIV-infected human macrophages through activating the Raf/ERK1/2 and AKT pathways (56–58). Cook et al. found that apelin/APJ signaling could prevent neuronal excitotoxic signaling by activating pro-survival pathways, including IP3, PKC, mitogen-activated protein kinase kinase 1/2 (MEK1/2), and ERK1/2, and concurrently by inhibiting NMDA receptor activity via regulating NMDA-induced ionic currents as well as Ca^2+^ accumulation, calpain activation, and NMDA receptor subunit NR2B phosphorylation at S1480 in cerebrocortical neurons (59). Similarly, studies from Zeng et al. indicated that NMDA-induced excitotoxicity in cortical neurons could be attenuated by apelin-13 (60). All of this evidence suggests that NMDA receptor can be used as a therapeutic target for ischemic stroke.

### Suppressing Oxidative and Nitrative Stresses

If the production of free radicals, mainly referring to reactive oxygen/nitrogen species (ROS/RNS), exceeds the intrinsic scavenging capacity of the antioxidative system, oxidative and nitrative stresses will occur, which play deleterious roles in cerebral ischemia (61–63). These stresses are part of the downstream consequences of neuronal excitotoxicity due to increased generation of free radicals via several oxidases, which is influenced by Ca^2+^ overload (64, 65). Substantial experiments indicate that the formation of free radicals increases in all types of stroke (66, 67).

Considerable research has shown that the apelin/APJ system can promote neuron survival by reducing oxidative and nitrative stresses. Apelin-13 can reduce I/R injury–induced oxidative stress by decreasing malondialdehyde (MDA) level and increasing superoxide dismutase (SOD) activity, which may be associated with the ERK1/2 signaling pathway (48). In a recent study, apelin-13 intervention significantly reduced the levels of ROS and MDA and increased the antioxidant proteins' expressions at the same time [glutathione (GSH), GSH-Px, catalase (CAT), and SOD] in a dose-dependent manner by activating adenosine monophosphate (AMP)-activated protein kinase (AMPK)/glycogen synthase kinase 3 β (GSK-3β)/nuclear factor erythroid 2–related factor 2 (Nrf2) signaling (52). The aforementioned evidence strongly suggests that the novel protective effect of the apelin/APJ system on cell death induced by oxidative stress may be achieved by inhibiting production of ROS and facilitating scavenging of ROS.

Nitric oxide (NO) plays dual roles in ischemic injury: when generated by eNOS, it exerts vasodilation and neuroprotective effects, but when produced by neuronal NOS (nNOS) and inducible NOS (iNOS), it is the main mediator of oxidative/nitrosative injury (61, 62, 68). Similarly, apelin has dual vascular effects. For example, activating the apelin/APJ axis induces endothelium- and NO-dependent peripheral arterial relaxation (69, 70). However, apelin/APJ signal can inhibit NO-induced cerebral artery relaxation by blocking calcium-activated K (BK_Ca_) channels in male rats, which can be mediated by a PI3K/AKT-dependent signaling pathway (71, 72). Meanwhile, the specific effect of apelin on oxidative/nitrosative stresses in ischemic stroke remains to be further determined.

### Inhibiting Inflammatory Responses

Inflammation plays a key role in the pathophysiological process of ischemic stroke, which may contribute to ischemic brain injury. Soon after the ischemic onset, inflammatory cells (e.g., microglia, astrocytes) are activated by multiple factors, including ROS, necrotic cells, and damaged tissues, which trigger inflammatory responses (73–76). Moreover, a number of studies have suggested that postischemic neuroinflammation plays a crucial role in the long-term prognosis of ischemia (77, 78).

The apelin/APJ system can inhibit inflammatory responses after ischemic stroke via reducing the generation of inflammatory mediators. For example, Chen et al. found that apelin-13 could reduce the expressions of chemokines and proinflammatory cytokines, including monocyte chemoattractant protein 1 (MCP-1), macrophage inflammatory protein 1α (MIP-1α), tumor necrosis factor α (TNF-α), and interleukin 1β (IL-1β), while the anti-apoptotic cytokine IL-10 was increased by apelin-13 in adult male C57/BL6 mice with ischemic stroke (79). Consistently, when compared with the I/R group, the expressions of many inflammatory cytokines such as TNF-α, IL-1β, and as well as intercellular adhesion molecule 1 (ICAM-1) are significantly reduced by apelin-13 treatment in a dose-dependent way, which is mediated by activating the AMPK/GSK-3β/Nrf2 pathway (12, 52). Additionally, apelin-13 remarkably decreases the activation and recruitment to the ischemic penumbra of microglia, which is related to the increase of cell survival (79). The most recent research indicates that apelin-13 ameliorated neuroinflammation by shifting N9 microglial M1 polarization toward the M2 phenotype, which may be related to the STAT3 signaling pathway (80). The above data indicate that apelin may be a potential target for regulating inflammation in ischemic stroke.

### Preventing ERS

Neuronal apoptosis induced by cerebral I/R injury includes many mechanisms, in which ERS and subsequent unfolded protein response (UPR) play important roles (13, 44, 81). Under conditions of ERS, glucose-regulated protein 78 (GRP78) is isolated from three membrane proteins, i.e., inositol-requiring enzyme 1 (IRE1), phosphorylation of protein kinase–like endoplasmic reticulum kinase (PERK), and ATF6, which initiates UPR, resulting in cell apoptosis (14, 44, 82).

Apelin gene expression is repressed by ATF4 via a p38 MAPK–dependent pathway under ERS (34). Meanwhile, apelin treatment can protect cells from apoptosis by preventing ERS induced by I/R injury in the brain (13, 14). For example, Qiu et al. reported that apelin-36 could inhibit the activation of ERS/UPR by markedly reducing CCAAT/enhancer binding protein homologous protein (CHOP) and GRP78 expression induced by cerebral I/R injury in the rat cortex (13). Moreover, apelin-13 can suppress eukaryotic translation initiation factor 2 (eIF2)-ATF4-CHOP–induced neuronal apoptosis via activating Gα_i_/Gα_q_-casein kinase 2 (CK2) signaling (14). Notably, ERS is initiated only in the reperfusion phase rather than in the ischemic phase, indicating that apelin intervention in the reperfusion phase may protect neurons from ERS-mediated apoptosis (42, 43).

### Modulating Autophagy

Like ERS, autophagy is also activated after ischemic stroke, and excessive ERS can induce autophagy and ultimately leads to neuronal apoptosis (83). Growing evidence indicates that the apelin/APJ system can inhibit apoptosis by regulating autophagy. First, apelin-13 pretreatment attenuates glucose deprivation-induced cardiomyocyte injury and decreases the autophagosome number and the ratio of microtubule-associated protein 1 light chain 3 (LC3)-II/LC3-I, which may be mediated by the PI3K/AKT/mechanistic target of rapamycin (mTOR) signaling pathway (84). Consistently, activating the PI3K/AKT/mTOR pathway by exogenous apelin can prevent hypoxia-induced autophagy and decrease cell proliferation and migration in an APJ receptor–dependent manner (85). In addition, apelin-13 suppresses traumatic brain injury (TBI) by decreasing autophagy-associated protein levels, such as LC3-II/I, Beclin-1, and Beclin-1/Bcl-2, which are expressed in the injured cortex and hippocampus in mice (86).

Up to now, there is no report about the role of apelin/APJ signaling in autophagy in ischemic stroke. However, the regulation of the apelin/APJ system in autophagy in the above diseases suggests that the apelin/APJ system may participate in autophagy induced by brain I/R injury.

### Promoting Angiogenesis

Post-stroke angiogenesis facilitates the recovery of cerebral blood flow (CBF) and the supply of substances and energy for neurogenesis (87). A number of studies have shown that vascular endothelial growth factor (VEGF), which is known to have neuroprotective effects in stroke, can improve the neuronal survival and the number of microvessels after cerebral ischemia (88–90). Chen et al. reported that apelin-13 could dramatically accelerate the expression of VEGF and matrix metalloproteinase-9 (MMP-9), which contribute to angiogenesis in mice with focal cerebral ischemic stroke (79). Recent studies suggest that apelin-13 protects the neurovascular unit against ischemic injuries, which is dependent on an increase of VEGF–VEGF receptor 2 (VEGFR2) signaling, possibly by activating ERK and PI3K/AKT pathways (51). Moreover, the apelin/APJ system may work in coordination with VEGF to trigger vascular sprouting and CBF recovery from human urinary kallidinogenase (HUK)–treated stroke patients in an ERK1/2-dependent manner (91, 92). On the contrary, the expression of VEGF in cultured endothelial cells is partially suppressed by the apelin/APJ system inhibitor, suggesting that the apelin/APJ system may cooperate with VEGF to promote vascular growth in ischemic stroke (92). Similarly, apelin deficiency causes compromised angiogenesis and functional recovery and increased susceptibility to ischemic injury (93). Apelin-13 protects the blood–brain barrier (BBB) from ischemic injury by upregulating aquaporin-4 (AQP4) and VEGF via the ERK and PI3K/AKT pathways (46). In conclusion, the apelin/APJ system not only protects neurons from ischemic injury but also facilitates angiogenesis and prognosis of stroke, which may be mediated by the VEGF–VEGFR2 signaling pathway.

## Relationship Between APELIN/APJ System Polymorphism and Susceptibility to Ischemic Stroke

Growing evidence has indicated that genetic factors exert important roles in stroke and that people with a family history of stroke are more likely to suffer from stroke (17, 94, 95). Data from genomewide association studies suggest that the heritability in all types of ischemic stroke is 37.9% (96). Hata et al. reported that rs9943582, a single-nucleotide polymorphism (SNP) which is located in the promoter region of the APJ gene and can enhance the expression level of APJ mRNA, could increase the risk of ischemic stroke in Japanese, and that the risk of ischemic stroke in the GG genotype was significantly higher than other genotypes (97–99). Meanwhile, there was no significant correlation between rs9943582 and ischemic stroke in the Chinese Han GeneID population in a case–control study including 1,158 patients and 1,265 controls (100). Consistently, in another study on the Chinese population from Zhang et al. no associations were detected between rs9943582 and the age of onset and prognosis of ischemic stroke (101). Further intensive study and repeated validation of the relationship between gene polymorphism of apelin/APJ system and ischemic stroke can predict the risk of ischemic stroke, which will provide strategies for prevention and treatment of ischemic stroke.

## Conclusions

The apelin/APJ system is widely distributed in the central nervous system, participating in many pathophysiological regulations of some brain diseases, including ischemic stroke (4, 15, 16). The apelin/APJ system shows a neuroprotective effect by blocking cell apoptosis or death and improving behavioral performance via various mechanisms including suppressing excitotoxicity, inflammatory responses, ERS, and oxidative and nitrative stresses, and at the same time modulating autophagy, promoting angiogenesis. Recently, researchers have found another endogenous ligand of APJ, apela, which enriches the functions of the system and complicates it as well (5, 6).

Ischemic stroke, known to be a devastating cerebrovascular disease with high morbidity, includes cerebral thrombosis, cerebral embolism, lacunar infarction, transient ischemic attack, and other types. The MCAO model is the most commonly used cerebral ischemia model (Table 1). Electroacupuncture-induced expression of apelin/APJ mRNA and protein of cerebral vascular endothelial cells in rats with cerebral infarction has an important role in the establishment of blood vessel regeneration and collateral circulation (102). In a transient model of focal cerebral ischemia, apelin-13 reduces brain injuries and postischemic cerebral edema in a dose-dependent manner, likely through inhibiting neuronal apoptosis, which may be mediated by many mechanisms, such as activating AMPK, PI3K/AKT, and ERK1/2 signaling pathways (47, 49, 50, 103). However, the specific effects of the apelin/APJ system on other types of ischemic stroke are rarely reported. And at the same time, due to the diversity of the active forms and signal pathways of apelin, it is a long way to go before the apelin/APJ system can be used in clinical practice. Further in-depth study of the physiological and pathological effects of the apelin/APJ system on ischemic stroke and the potential mechanisms will help to guide clinical prevention of and intervention in ischemic stroke and develop a series of drugs targeting different subtypes and phases of the disease, providing good news for patients, which will reduce family and social burdens.

## Author Contributions

YT, RC, YJ, and BB wrote the manuscript. TY and HL formulated and revised the manuscript.

### Conflict of Interest

The authors declare that the research was conducted in the absence of any commercial or financial relationships that could be construed as a potential conflict of interest.
